# Daily Consumption of Chocolate Rich in Flavonoids Decreases Cellular Genotoxicity and Improves Biochemical Parameters of Lipid and Glucose Metabolism

**DOI:** 10.3390/molecules23092220

**Published:** 2018-09-01

**Authors:** Aldo Leyva-Soto, Rocio Alejandra Chavez-Santoscoy, Linda Ramona Lara-Jacobo, Ana Vianey Chavez-Santoscoy, Lina Natalia Gonzalez-Cobian

**Affiliations:** 1 Facultad de Ciencias Químicas e Ingeniería, Universidad Autónoma de Baja California—Campus Tijuana, Calzada Universidad 14418, Parque Industrial Internacional Tijuana, C.P. 22390 Tijuana, Mexico; aldo.leyva@uabc.edu.mx (A.L.-S.); ngonzalez8@uabc.edu.mx (L.N.G.-C.); 2 Laboratoire Écotoxiquegénomique, Eau Terre Environment, Institut national de la Recherche Scientifique (INRS-ETE), Rue 490 Couronne, Quebec City, QC G1K 9A9, Canada; linda_ramona.lara-jacobo@ete.inrs.ca; 3 Department of Cellular and Structural Biology, University of Texas Health Science Center at San Antonio, 7703 Floyd Curl Drive, San Antonio, TX 78229, USA; anayenaiv@gmail.com

**Keywords:** flavonoids, dark chocolate, genotoxicity, lipid metabolism, glucose metabolism

## Abstract

In recent years, the incidence of atherosclerotic cardiovascular disease, obesity, and diabetes has increased largely worldwide. In the present work, we evaluated the genoprotective effect of the consumption of flavonoids-rich chocolate on 84 young volunteers. Biochemical indicators related to the prevention and treatment of cardiovascular risk and metabolic syndrome were also determined. A randomized, placebo-controlled, double-blind study was performed in the Autonomous University of Baja California. The treatments comprised the daily consumption of either 2 g of dark chocolate containing 70% cocoa, or 2 g of milk chocolate, for 6 months. The total amount of phenolic compounds and flavonoids was determined in both types of chocolate. Anthropometrical and Biochemical parameters were recorded prior to and after the study. The evaluation of the genotoxicity in buccal epithelial cells was performed throughout the duration of the study. Flavonoids from cocoa in dark chocolate significantly prevented DNA damage, and improved the nucleus integrity of cells. This effect could be related to the antioxidant capacity of the dark chocolate that decreased cellular stress. Biochemical parameters (total cholesterol, triglycerides, and LDL-cholesterol level in blood) and anthropometrical parameters (waist circumference) were improved after six months of daily intake of 2 g of dark chocolate with a 70% of cocoa.

## 1. Introduction

Obesity is a rising global health problem that affects 600 millions of adults worldwide [1]. The prevalence of obesity is directly related to the development of cardiovascular diseases and diabetes through a condition known as metabolic syndrome [2,3]. Atherosclerotic Cardiovascular Disease (ACVD) is the leading cause of death around the world [1]. High levels of LDL and triglycerides, and low levels of HDL are associated with the development of ACVD [4]. Metabolic Syndrome is a condition that leads to other mortal conditions such as hypertension, dyslipidemia, ACVD, and diabetes [5,6,7]. The incidence of all these pathologies is increasing worldwide; therefore, they are becoming a public health concern.

Diet is one of the major lifestyle factors that can significantly influence the incidence and progression of chronic diseases such as cardiovascular disease, diabetes, and cancer. Humans have consumed cocoa since at least 460 AD [8]. An increasing number of reports have shown that the consumption of cocoa and dark chocolate exerts several beneficial effects on cardiovascular health and endothelial dysfunction [9]; observations are consistent with reports that dark chocolate lowers blood pressure [9] and improves endothelium-dependent vasodilator responses [10]. Cocoa, especially dark chocolate, contains high levels of flavanols such as epicatechin, catechin, and procyanidins. Flavanols in cocoa are present as either the monomers (−) epicatechin and (+) catechin or oligomers of epicatechin and/or catechin, called proanthocyanidins or condensed tannins. Many beneficial effects have been attributed to flavonoids. For example, some reports have shown that epicatechins improved vascular function, reduced BP, improved insulin sensitivity, and reduced platelet activity [11]. Flavonoid content in chocolate varies among brands due to differences in the production process. The initial levels of flavonoids in the cocoa bean could change during fermentation, drying, roasting, alkalinization, and storage conditions, depending on the protocols of each company; even if the percentage of cocoa is the same, the final content of flavonoids might be different. Moreover, there is no specific regulation governing the labeling of dark chocolate or of its polyphenol or flavonoid content [12].

In metabolic syndrome, patients present a chronic systemic inflammation condition which has been widely related to cellular stress [2]. It is also known that cellular stress is related to the propensity to generate genetic mutation and cancer [13]. In recent years, increasing attention has been given to compounds and conditions that induce genetic damage by various mechanisms. Specifically, the mutagenic events and genotoxic agents might play an important role in the cause and/or progression of human diseases other than cancer [14]. Physical activity and nutrition are important modifiers of systemic oxidative stress. The most reported metabolic disease that has been associated with DNA damage is diabetes. DNA damage is associated with diabetes; the extent of damage is greater in diabetics compared to non-diabetics [15] Interestingly, it has been reported that systemic DNA damage has a significant correlation with elevated parameters of metabolic syndrome [16]. Recently, oral epithelium cells have been used for the evaluation of exposure to various genotoxic agents, associated with its recognized sensitivity for the assessment of DNA damage. The micronucleus test is a multi-target genotoxic endpoint. Therefore, it can provide additional insight about epigenetic effects in different cell types caused by an agent or a condition. Thus, the determination of abnormalities in buccal epithelial cell nuclei has been used as a noninvasive assay for monitoring genotoxicity, and hence, DNA damage in humans.

In the present work, we evaluate the genoprotective effect of consuming a flavonoids-rich chocolate, and the improvement in the biochemical parameters related to cardiovascular risk and metabolic syndrome in young Mexican adults.

## 2. Results

### 2.1. Study Population

Table 1 describes participant characteristics in terms of sex, education level, physical activity, and cardiometabolic co-morbidities (diabetes and hypertension). Physical activity was classified as inactive, moderately active, and active, and it was assessed according to the International Physical Activity Questionnaire of World Health Organization [17]. The majority of the studied population was undergraduate students, with normal blood pressure but high triglyceride and glucose levels (Table 1). Participants presented an average body mass index (BMI) of 32.1, with an incidence of approximately 68% (Table 1). According to the World Health Organization, for adults over 20 years of age, a BMI ranging from 30.0 to 34.9 falls within the Obesity Class I range [18]. The characteristics of each intervention group are described in Table 1. Since a randomized study was conducted, characteristics are similar in both groups.

### 2.2. Determination of Total Phenolic, Flavonoids Content and Antioxiant Capacity

Total phenolics content was quantified in commercial dark chocolate and milk chocolate (Table 2). Total phenolic content was 63.7 ± 0.2 and 56.3 ± 1.5 µmol of gallic acid equivalents per 1 g of dark chocolate and milk chocolate respectively. Dark chocolate and milk chocolate had 34.8 ± 0.5 and 10.4 ± 0.8 µmol of (−)-catechin equivalents per 1 g respectively. The percentage of flavonoids/phenolics (%) was significantly higher in dark chocolate than in milk chocolate. The antioxidant capacity (Table 2) was significantly greater in dark chocolate than in milk chocolate (*p* < 0.05).

### 2.3. Changes Caused by Dailyconsumption of Chocolate

The dietary variables did not change as a consequence of the consumption of flavonoid-rich chocolate. In contrast, some anthropometric and biochemical variables varied after the dietary intervention with dark chocolate (Table 2). Variables were evaluated before and after the treatment of either milk or dark chocolate daily consumption for 6 months.

### 2.4. Frequency of Nuclear Abnormalities in Buccal Epithelial Cells

**Figure 1:**
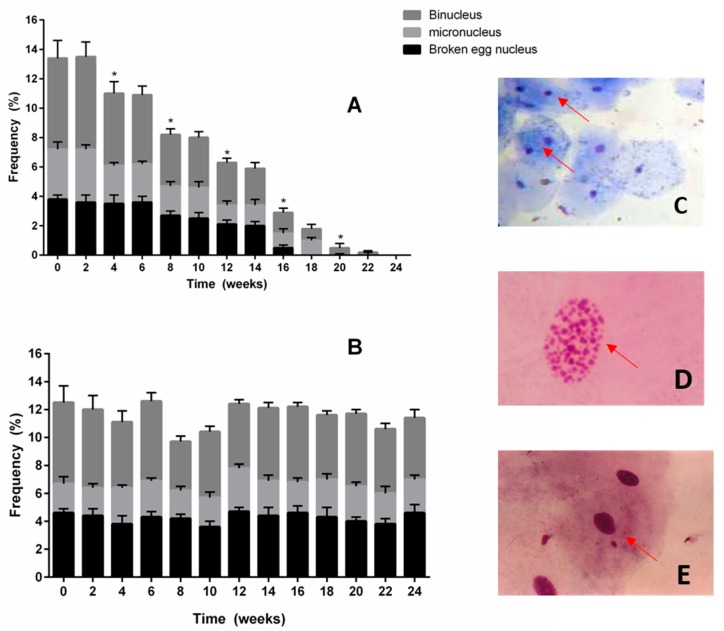
Frequency in nuclear abnormalities in buccal epithelial cells during consumption of dark chocolate with 70% cocoa (**A**) and milk chocolate (**B**). * Frequency was stadistically compared with the frequency obtained in the previous week (*p* < 0.05). Observed nuclear abnormalities were (**C**) binucleus (**D**) micronucleus, and (**E**) broken egg.

At the beginning of the study, 14.4% of the buccal epithelial cells had abnormalities of the nuclei. Likewise, participants of both experimental groups showed nucleus abnormalities of broken egg, micronucleus, and binucleus at the beginning of the study (Figure 1). Interestingly, abnormalities of the nuclei in the buccal epithelial cells decreased significantly (less than 2%) after 6 month of daily consumption of 2 g of dark chocolate (Figure 1A). Milk chocolate intake (2 g per day for 6 months) did not affect the percentage of abnormalities observed in buccal epithelial cells.

**Figure 2:**
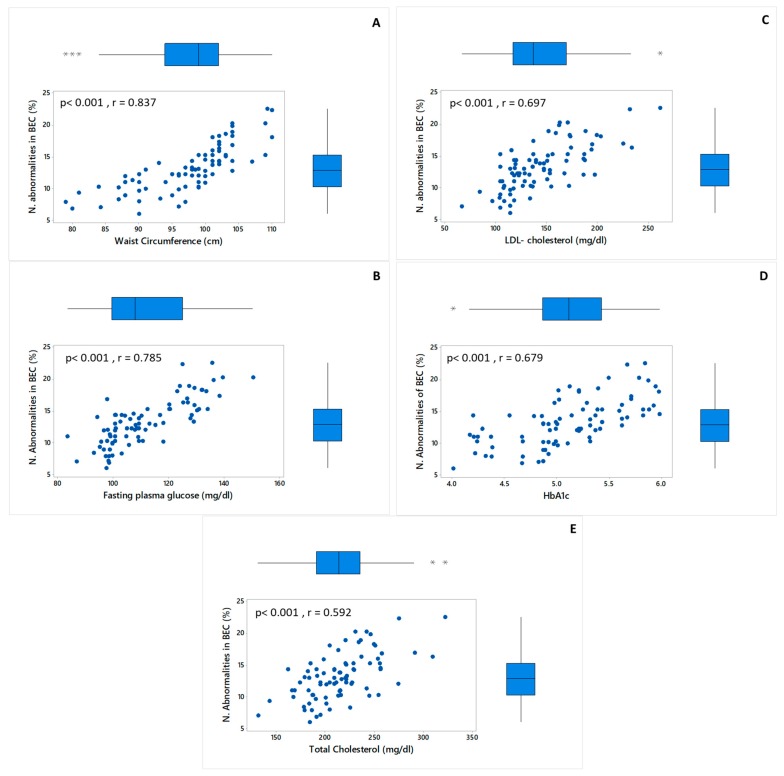
Correlation study through Pearson’s linear regression analysis in the beginning of the study at the 84 participants. (**A**) Correlation between nuclear abnormalities in bucall epithelial cells (N. Abnormalities in BEC) and waist circumference (*p* < 0.001, *r* = 837). (**B**) Correlation between nuclear abnormalities in bucall epithelial cells (N. Abnormalities in BEC) and fasting plasma glucose (*p* < 0.001, *r* = −0.785). (**C**) Correlation between nuclear abnormalities in bucall epithelial cells (N. Abnormalities in BEC) and LDL-cholesterol levels (*p* < 0.001, *r* = 0.697). (**D**) Correlation between nuclear abnormalities in bucall epithelial cells (N. Abnormalities in BEC) and HbA1c (*p* < 0.001, *r* = −0.679). (**E**) Correlation between nuclear abnormalities in bucall epithelial cells (N. Abnormalities in BEC) and total-cholesterol levels (*p* < 0.001, *r* = 0.592). Atypical points were marked with *.

The Correlation study through Pearson’s linear regression analysis among the frequency of nuclear abnormalities in buccal epithelial cells and biochemical and anthropometrical variables was determined. The correlation study was performed with the biochemical and anthropometrical levels of the 84 participants at the beginning of the study. Figure 2 shows the parameters that had a significant correlation (r ≥ 0.5, *p* < 0.001) with nuclear abnormalities in buccal epithelial cells. Waist circumference (*r* = 837, *p* < 0.001) and Fasting glucose plasma (*r* = 785, *p* < 0.001) were the parameters with highest correlations with the frequency of nuclear abnormalities in buccal epithelial cells.

## 3. Discussion

### 3.1. Determination of Total Phenolic and Flavonoids Content

Results showed that 54.6% of phenolic content in the dark chocolate was from the flavonoids group; this percentage of flavonoids was significantly higher compared with the 18% of flavonoids contained in the milk chocolate. The relation flavonoids/polyphenols showed that the dark chocolate with 70% cocoa was a significantly better source of flavonoids than milk chocolate. Taking into consideration that 2 g of dark chocolate were provided to each participant in the study, approximately 69.6 µmol of (-)-catechin equivalents of flavonoids were provided to each participant daily in the treatment with commercial dark chocolate with 70% cocoa (FRC), and 20.8 µmol of (−)-catechin equivalents of flavonoids were provided to each participant in the milk chocolate treatment (MC).

### 3.2. Characteristics of Study Population

A total of 84 participants were recruited for the present study. The majority of the participants were undergraduate students (94%), of which 38% were current smokers, 65% reported having a habit of moderate physical activity, and 67% presented a BMI in a range of 30–34.9 (falling into the Obesity class I range). Half of the participants (*n* = 42) were randomly included in the treatment group where commercial dark chocolate with 70% cocoa was provided, and the other half were treated with milk chocolate. The principal reported risk factor by participants was dyslipidemia, as 61.90% of participants presented at least one of the following anomalies: total cholesterol ≥190 mg/dL (≥4.9 mmol/L), TAG ≥150 mg/dL (≥1.7 mmol/L), LDL-cholesterol ≥115 mg/dL (≥3.0 mmol/L), HDL-cholesterol <40 mg/dL for men and <46 mg/dL for women.

### 3.3. Determination of Total Phenolic and Flavonoids Content

According to the results, the content of phenolics was slightly lower in milk chocolate compared to dark chocolate; however it has been reported that sugar content could affect the measurement of phenolic content by Folin-Ciocalteu method. Interestingly, the flavonoid content was 3 fold higher in the dark chocolate compared with the milk chocolate used for the present study. Since the percentage of flavonoids/phenols (%) was significantly higher in dark chocolate than milk chocolate, the commercial dark chocolate used as a treatment was a better source of flavonoids compared with the commercial milk chocolate used in the present study.

There was no significant difference in the intake of fruits and vegetables reported by the participants (Table 3). However, a significant amount of flavonoids were provided on a daily basis by dark chocolate intake (Table 2), comparing the content of flavonoids of dark and milk chocolates (Table 2). Flavonoids are the most bioactive molecules reported among polyphenolic compounds. The flavonoids/phenols ratio shows the proportion of total polyphenolic compounds that belong to flavonoids. According to results, 54% of total phenolic compounds of dark chocolate are flavonoids, while only 18% of total phenolic compounds are flavonoids in milk chocolate. It has been reported that the most abundant flavonoid found in cocoa is the flavonol epichatechin, followed by chatechin. Also, the authors reported that some dimers and polymers of those flavonols were found in most of analyzed chocolates [12].

The antioxidant capacity was 3-fold greater in dark chocolate than in milk chocolate (Table 2). The antioxidant capacity has been previously related to the content of phenolic and flavonoid compounds. Moreover, the antioxidant capacity of cocoa might be higher than red wine and black tea [19].

### 3.4. Changes in Measured Parameters by Daily Dark Chocolate Consumption

Dietary variables did not significant change by the consumption of flavonoid-rich chocolate. However, certain anthropometric and biochemical variables varied after consumption of the flavonoid-rich chocolate (Table 2). The waist circumference of participants was significant lower (*p* < 0.05) after the study, a change that could not be attributed to changes in diet, since Total energy intake, and the proportion of carbohydrates and lipids were similar before and after the study. Interestingly, total blood cholesterol, triglycerides, and LDL-cholesterol significantly decreased after six months of daily intake of dark chocolate with high content of flavonoids (*p* < 0.05). It has been previously reported that dark chocolate intake could improve LDL levels in blood [20]. Moreover, it was also reported that the consumption of flavonoids could significantly increase lipid oxidation [21,22]; thus, flavonoids intake might improve lipid biochemical parameters by the modulation of reverse cholesterol transport in gut and liver [23]. With regards to HOMA-IR and fasting plasma glucose, both parameters were significantly decreased after 6 months of daily intake of the flavonoid-rich chocolate. HOMA-IR is a homeostatic model assessment (HOMA) to determine insulin resistance (IR) in β-cells. HOMA-IR is calculated with the proportion of plasma glucose and insulin levels. According to the results of the present study, and in accordance with previous reports [24], daily flavonoid-rich chocolate intake was significantly associated with a lower HOMA-IR (*p* < 0.05). Flavonoids such as genistein and epicatechin have been previously associated with a lowering of blood glucose concentration [21,25]. Finally, and no less important, blood pressure was significantly improved by the consumption of flavonoid-rich chocolate compared with milk chocolate (Table 3). Cocoa flavonoids have been previously reported for their effect in the improvement of cardiovascular parameters, such as blood pressure and platelet aggregation [26].

### 3.5. Frequency of Nuclear Abnormalities in Buccal Epithelial Cells

The abnormalities of the nuclei in the buccal epithelial cells were 14.4% at the beginning of the study. The participants of both experimental groups showed abnormalities of broken egg nucleus, micronucleus, and binucleus at the beginning of the study (Figure 1). Interestingly, waist circumference (*p* < 0.001, *r* = 837) and fasting plasma glucose (*p* < 0.001, *r* = −0.785) showed most significant correlation with the frequency of nuclear abnormalities in buccal epithelial cells at the beginning of the study (Figure 2). The results of the present study suggested that the risk factors of metabolic syndrome could be associated with the development of genomic instability (Figure 2). Other authors have shown an association of diabetic patients in the highest percentile of waist circumference, fasting plasma glucose, HbA1c, and cardiovascular risk with buccal-epithelia-cells increased genomic instability [27]. The exact mechanism that contributes to this genomic instability in obesity and metabolic syndrome is not clear, although the primordial link between them is oxidative stress, caused by chronic and systemic inflammation [2]. The increase in DNA damage might be occurring because of the increase in the imbalance between the production of oxidants and antioxidant defenses [28]. Moreover, epidemiologic studies have associated DNA damage with obesity and diabetes [15,27,29]. However, more in-depth studies should be conducted to determine the relationship between genomic instability and conditions of metabolic syndrome.

It was expected that nuclear abnormalities in buccal epithelial cells of participants would be found, because most of them presented risk factors that have been related with genotoxicity, such as the habit of smoking, obese state, and high glucose levels in plasma [30]. One of the most important findings of this study is that the daily consumption of 2 g of dark chocolate for 6 months could significantly decrease the genotoxicity and cellular damage of buccal epithelial cells. In particular, the frequency of nuclear abnormalities significantly decreased with the daily consumption of flavonoid-rich chocolate compared to milk chocolate intake. This might be the result of flavonoids increasing the antioxidant activity in the cells of the participants, and as a consequence, the DNA damage in buccal epithelial cells significantly decreased. The antioxidant capacity of flavonoids in dark chocolate (Table 2) could be related to its protective cellular effect, that decreases cellular stress, and hence, DNA damage (Figure 1). This effect was a novel finding, because flavonoid consumption in dark chocolate has not been related with antigenotoxic effect before. The decreasing effect of genotoxicity might be related to the decreasing effect of oxidative stress in cells due to the antioxidant capacity and activity of flavonoids, and the modulation of expression of CYP450 [31]. Other genotoxic assays should be conducted to determine if the protective effect of flavonoids obtained in the present study is a systemic effect.

Interestingly, some studies have reported that Orlistat, an antiobesty drug, induced DNA damage in human cells, which suggested that regular consumption of orlistat needs careful circumspection [32]. In contrast, flavonoids from cocoa in dark chocolate are able to significantly decrease important risk factors such as waist circumference, total cholesterol, LDL-cholesterol, and triglycerides, without causing DNA damage, even having a celluloprotective effect that could reverses the DNA damage in buccal epithelial cells, which is apparently caused by the systemic inflammation of obesity, along with the fact that flavonoids have been long recognized for their anti-inflammatory effect [33].

## 4. Materials and Methods

### 4.1. Study Design and Participants

A randomized, placebo-controlled, double-blind study was performed in the Autonomous University of Baja California. The study was approved by the Ethics Committee of faculty of Medicine and Psychology of Autonomous University of Baja California (1120150617), and written consent was obtained from all subjects prior to enrollment in the study.

In brief, a random sample was recruited between November 2015 and January 2018. The inclusion criteria for the enrollment in the study were (1) Mexican Nationality with parents of Mexican Nationality–Hispanic Ethnicity; (2) Being from 20 to 35 years old; and (3) Having at least 3 of 5 risk factors: Glucose greater than 100 mg/dL, triglycerides levels greater than 160 mg/dL, LDL greater than 130 mg/dL, HDL lower than 45 mg/dL, and body mass index (BMI) greater than 29. Exclusion criteria for participation in the study were: taking antihypertensive, hypocholesterolemic, or weight-loss medications, or taking any chocolate or cocoa extract regularly. The elimination criteria were to start taking antihypertensive, hypocholesterolemic, weight-loss medications, or any chocolate or cocoa extract during the experiment, or simply to decide to leave the study. Trained research staff provided the participants with detailed instructions for the study, assisted them in completing questions on dietary information, and then checked the completeness and accuracy of the responses. The dietary intervention was carried out during 6 months with a weekly follow-up of the participants. During the weekly interviews, a dietary survey was carried out; questions of the study were answered, and it was verified that the participants did not fall into the selimination criteria. The study began with 92 participants after data cleaning, particularly for poorly completed dietary data. During the study, 8 people were removed because of elimination criteria; the rest finished the study (*n* = 84).

### 4.2. Chocolate Consumption (Independent Variable)

The commercial dark chocolate of 70% cocoa content or milk chocolate was provided to participants by the research staff weekly, packaged in daily portions; the daily dose for each patient was 2 g of chocolate. The participants were blinded because the original packaging of the chocolates was removed; the chocolates were divided into 2 g portions, and packed again inside a white envelope. A weekly semi-quantitative survey was completed by the participants, including questions on habitual daily consumption of chocolate during the previous week. The participants reported their frequency of consumption, ranging from 1 to 7 days of consumption. They also selected the serving size based on how many packaged portions were ingested every day.

### 4.3. Determination of Total Phenolic Content

Total phenolic content determination was made using the Folin-Ciocalteu method [34], Briefly, extraction was performed with 80% methanol in water (*v/v*), then total polyphenols content was determined by using Beckman Coulter DU 520 Spectrophotometer (Beckman, Carlsbad, CA, USA) at 750 nm. A standard curve was developed using Gallic acid (Sigma-Aldrich, Lansing, MI, USA).

### 4.4. Determination of Flavonoids Content

Flavonoids were evaluated in 80% methanol in water (*v/v*) extracts with a colorimetric method, according to previous reports [34], using a Coulter DU 520 Spectrophotometer at 510 nm. A standard curve was developed with (−)-catechin (Sigma-Aldich).

### 4.5. Antioxidant Capacity

The capacity of dark and milk chocolates to scavenge the free radical cation of diammonium salt (ABTS^+^) was conducted as reported in [19]. Briefly, Trolox standard curves were used to measure the capacity to diminish free radicals of diammonium salt (ABTS) radical solution. Absorbance was measured using a Coulter DU 520 Spectrophotometer at 734 nm.

### 4.6. Epithelial Genotoxicity, Biochemical and Anthropometric Parameters Measure (Dependent Variables)

After 4 weeks of daily consumption of flavonoid-rich chocolate, a blood sample was obtained for each participant in order to measure biochemical parameters. Standard laboratory assays were used to measure Biochemical parameters with IDEXX Catalyst Dx^®^ equipment (IDEXX, Westbrook, ME, USA). Anthropometric parameters were also measured every 4 weeks with a digital weighing scale and a measuring tape.

Genotoxicity was determined in each participant by a micronuclei assay. Briefly, exfoliated cells were collected by a non-invasive sampling method from oral mucosa. Buccal cavity cells are obtained by scraping the cheeks with a tongue depressor. The samples were transferred dropwise to pre-cleaned slides. Then, the slides were air-dried and fixed with 80% methanol. After that, slides were stained with hematoxylin for 3–5 min, and then with eosin for 5–8 min. Genotoxic damage was determined by the observation of slides under the microscope; cellular malformations were observed and counted.

### 4.7. Statistical Analyses

Data were analyzed by using MiniTab and GraphPad Prism (version 6, GraphPad Software Inc., La Jolla, CA, USA) software. Results are reported as mean ± SEM. Statistical differences for mean data obtained from present study were analyzed by two-way ANOVA. Correlation study through Pearson’s linear regression analysis was made using MiniTab 18.

## 5. Conclusions

In conclusion, flavonoids of cocoa had protective effects against DNA damage. This suggested that the reduction in genotoxic stress effect was related with the antioxidant activity of flavonoids and the modulation of CYP450. The modulation of CYP450 and the reduction in the expression of some interleukins have been related with the anti-inflammatory effect of flavonoids [33]. The antioxidant capacity of flavonoids contained in dark chocolate could be related to the decrease of cellular stress, and hence, to the DNA protection in the nucleus of cell. However, further in vivo studies are still needed to determine their mechanism of action of antigentotoxic effect. Biochemical parameters (total cholesterol, triglycerides, and LDL-cholesterol level in blood) and anthropometrical parameters (waist circumference) were also improved after six months of dark chocolate with a 70% of cocoa intake. Interestingly daily flavonoid-rich chocolate intake also improves fasting plasma glucose levels and insulin resistance parameter (HOMA-IR). These effects were attributed to the proportion of flavonoids in the chocolate which was 3-fold greater than in the milk chocolate. Together, these results suggested a potential beneficial effect as a consequence of the daily dark chocolate consumption in the lipid and glucose metabolism.

## Figures and Tables

**Table 1:** Characteristics of study population at recruitment.

Characteristics		*n*	(%)	FRC (*n*)	MC (*n*)	Observations
Participants		84	(100)	42	42	
Sex	Men	47	(55.90)	23	24	
	Women	37	(44.10)	19	18	
Education level (completed or in progress)						According to the information provided in the surveys conducted.
Elementary school		2	(2.38)	1	1	
High school		3	(3.57)	2	1	
University		79	(94.05)	39	40	
Current smokers		32	(38.09)	16	16	
Physical activity						According to the information provided in the surveys conducted.
Inactive		17	(20.24)	8	9	
Moderate active		55	(65.48)	28	27	
Active		12	(14.28)	6	6	
Obesity (BMI range)						The classification was made according to of the world health organization (WHO)
Underweight (Below 18.5)		0	(0.00)	0	0	
Normal weight (18.5–24.9)		9	(10.71)	4	5	
Pre-obesity (25.0–29.9)		10	(11.90)	5	5	
Obesity class I (30.0–34.9)		57	(67.85)	29	28	
Obesity class II (35.0–39.9)		8	(9.52)	4	4	
Obesity class III (Above 40)		0	(0.00)			
Risk Factors	Hypertension	18	(21.41)	9	9	Defined as blood pressure ≥140 mm Hg and/or ≥90 mm Hg for systolic and diastolic pressures respectively.
	Diabetes	12	(14.28)	6	6	Defined as taking antidiabetic medications and/or having fasting plasma glucose ≥120 mg/dL (≥7 mmol/L)
	Dyslipidemia	52	(61.90)	26	26	Defined as having at least one of the following anomalies: total cholesterol ≥190 mg/dL (≥4.9 mmol/L), TAG ≥150 mg/dL (≥1.7 mmol/L), LDL-cholesterol ≥115 mg/dL (≥3.0 mmol/L), HDL-cholesterol <40 mg/dL for men and <46 mg/dL for women.

FRC = commercial dark chocolate with 70% cocoa, MC = commercial milk chocolate.

**Table 2:** Phenolic and Flavonoid content in used dark and milk chocolates for the study.

Treatment	Phenolic Content (µmol Gallic Aid Equivalents/g)	Flavonoid Content (µmol Catechin Equivalents/g)	Antioxidant Capacity (µmol Trolox Equivalents/g)	Flavonoids/Phenolics (%)
FRC	63.70 ± 0.20	34.80 ± 0.50	3.52 ± 0.60	54.63
MC	56.30 ± 1.50	10.40 ± 0.80	0.98 ± 0.02	18.47

FRC = commercial dark chocolate with 70% cocoa, MC = commercial milk chocolate.

**Table 3:** Dietary, anthropometric and biochemical variables determined prior and after the dietary intervention with either flavonoid-rich chocolate or milk chocolate for 6 months.

Variables		Intervention Group	Beginning of the Study Mean ± SD	End of the Study Mean ± SD
Age		FRC MC	23.8 ± 3.4 23.6 ± 3.5	24.6 ± 3.1 23.8 ± 2.6
Number of Participants		FRC MC	42 42	42 42
Dietary variables	Fruit and vegetable intake (g/day)	FRC MC	523.7 ± 371.4 529.1 ± 329.6	548.2 ± 387.9 531.2 ± 356.21
	Total energy intake (kJ/day)	FRC MC	2214 ± 323 2208 ± 350	2298 ± 236 2310 ± 120
	Total carbohydrate (%E)	FRC MC	45.3 ± 8.2 46.7 ± 6.3	43.8 ± 8.9 45.8 ± 7.2
	Added sugar	FRC MC	5.3 ± 3.2 5.8 ± 4.1	6.1 ± 2.7 5.8 ± 3.8
	Total Fat (%E)	FRC MC	36.9 ± 8.1 34.8 ± 6.2	37.1 ± 7.6 36.5 ± 5.1
	Saturated Fat (%E)	FRC MC	20.8 ± 6.1 21.2 ± 3.1	16.9 ± 6.9 22.3 ± 5.9
	Unsaturated Fat (%E)	FRC MC	14.6 ± 4.1 14.5 ± 3.9	14.8 ± 4.6 15.9 ± 3.1
Anthropometric variables	BMI (Kg/m^2^)	FRC MC	32.1 ± 3.8 31.4 ± 3.2	30.1 ± 2.2 32.4 ± 2.5
	Waist Circumference (cm)	FRC MC	98.7 ± 3.5 96.9 ± 4.1	**90.4 ± 4.5** * 94.9 ± 3.9
Biochemical variables	Total Cholesterol (mg/dL)	FRC MC	221.3 ± 16.7 224.3 ± 18.9	**201.2 ± 19.5** * 227.4 ± 12.4
	LDL-Cholesterol (mg/dL)	FRC MC	149.82 ± 18.4 147.23 ± 21.1	**116.2 ± 21.1** * 138.9 ± 19.1
	HDL-Cholesterol (mg/dL)	FRC MC	46.3 ± 12.5 45.4 ± 12.1	43.2 ± 10.9 44.2 ± 13.5
	Triglycerides (mg/dL)	FRC MC	228.25 ± 17.9 223.5 ± 21.1	**153.26 ± 18.95** * 224.1 ± 23.1
	HOMA-IR	FRC MC	2.3 ± 1.8 2.5 ± 1.6	**1.93 ± 1.1** * 2.4 ± 1.5
	Fasting plasma glucose (mg/dL)	FRC MC	114.23 ± 13.56 112.31 ± 16.71	**91.23 ± 9.25** * 111.67 ± 10.9
	HbA1c (%)	FRC MC	5.8 ± 1.0 4.7 ± 1.0	4.6 ± 1.1 4.5 ± 0.9
	Systolic blood pressure (mmHg)	FRC MC	139.2 ± 10.5 136.3 ± 21.5	**127.8 ± 11.2** * 133.9 ± 12.7
	Diastolic blood pressure (mmHg)	FRC MC	87.24 ± 11.8 87.28 ± 9.18	**84 ± 9.12** * 87.31 ± 9.44

FRC = commercial dark chocolate with 70% cocoa, MC = commercial milk chocolate. * *p* values for testing the differences among variables across two groups (before and after chocolate consumption) by using X^2^ test. *p* < 0.05.
